# Supplementary material for: Nucleotide imbalance decouples cell growth from cell proliferation
Source: Nat Cell Biol. 2022 Aug 4;24(8):1252–64. doi: 10.1038/s41556-022-00965-1 (PMC9359916; doi:10.1038/s41556-022-00965-1)
Supplement: Supplementary file 1 — Reporting Summary [file 41556_2022_965_MOESM1_ESM.pdf]

nature portfolio

Corresponding author(s): Matthew Vander Heiden

Last updated by author(s): May 26, 2022

Reporting Summary

Nature Portfolio wishes to improve the reproducibility of the work that we publish. This form provides structure for consistency and transparency in reporting. For further information on Nature Portfolio policies, see our Editorial Policies and the Editorial Policy Checklist.

Statistics

For all statistical analyses, confirm that the following items are present in the figure legend, table legend, main text, or Methods section.

n/a	Confirmed	
☐	☒	The exact sample size (*n*) for each experimental group/condition, given as a discrete number and unit of measurement
☐	☒	A statement on whether measurements were taken from distinct samples or whether the same sample was measured repeatedly
☒	☐	The statistical test(s) used AND whether they are one- or two-sided<br>*Only common tests should be described solely by name; describe more complex techniques in the Methods section.*
☒	☐	A description of all covariates tested
☒	☐	A description of any assumptions or corrections, such as tests of normality and adjustment for multiple comparisons
☐	☒	A full description of the statistical parameters including central tendency (e.g. means) or other basic estimates (e.g. regression coefficient) AND variation (e.g. standard deviation) or associated estimates of uncertainty (e.g. confidence intervals)
☒	☐	For null hypothesis testing, the test statistic (e.g. *F*, *t*, *r*) with confidence intervals, effect sizes, degrees of freedom and *P* value noted<br>*Give P values as exact values whenever suitable.*
☒	☐	For Bayesian analysis, information on the choice of priors and Markov chain Monte Carlo settings
☒	☐	For hierarchical and complex designs, identification of the appropriate level for tests and full reporting of outcomes
☒	☐	Estimates of effect sizes (e.g. Cohen's *d*, Pearson's *r*), indicating how they were calculated

Our web collection on statistics for biologists contains articles on many of the points above.

Software and code

Policy information about availability of computer code

Data collection	BD FACSDiva v9.0 software was used to collect flow cytometry data. Live cell imaging data was collected using IncuCyte S3 2017A live cell imaging system (Sartorius).
Data analysis	Flow cytometry data was analyzed using FlowJo v10 software. Mass spectrometry data was analyzed with XCalibur QuanBrowser 2.2. Comet assay data was anlyzed with ImageJ with OpenComet v1.3 software.

For manuscripts utilizing custom algorithms or software that are central to the research but not yet described in published literature, software must be made available to editors and reviewers. We strongly encourage code deposition in a community repository (e.g. GitHub). See the Nature Portfolio guidelines for submitting code & software for further information.

Data

Policy information about availability of data

All manuscripts must include a data availability statement. This statement should provide the following information, where applicable:

- Accession codes, unique identifiers, or web links for publicly available datasets
- A description of any restrictions on data availability
- For clinical datasets or third party data, please ensure that the statement adheres to our policy

Numerical source data and unprocessed blots are available in the source data files for their corresponding figures.

Field-specific reporting

Please select the one below that is the best fit for your research. If you are not sure, read the appropriate sections before making your selection.

☒ Life sciences ☐ Behavioural & social sciences ☐ Ecological, evolutionary & environmental sciences

For a reference copy of the document with all sections, see nature.com/documents/nr-reporting-summary-flat.pdf

Life sciences study design

All studies must disclose on these points even when the disclosure is negative.

Sample size	Experiments were performed using standard sample sizes that produced tolerable error with respect to effect size. No statistical test was performed to predetermine sample size.
Data exclusions	No data was excluded.
Replication	Experiments were repeated in biological triplicates.
Randomization	This is not relevant to the study, as all experiments were done using human cell lines. No experiments involved allocation of different samples, organisms, or participants into experimental groups.
Blinding	Blinding was not relevant to the study because no experiments involved allocation of different samples, organisms, or participants into experimental groups.

Reporting for specific materials, systems and methods

We require information from authors about some types of materials, experimental systems and methods used in many studies. Here, indicate whether each material, system or method listed is relevant to your study. If you are not sure if a list item applies to your research, read the appropriate section before selecting a response.

Materials & experimental systems

n/a	Involved in the study	
☐	☒	Antibodies
☐	☒	Eukaryotic cell lines
☒	☐	Palaeontology and archaeology
☒	☐	Animals and other organisms
☒	☐	Human research participants
☒	☐	Clinical data
☒	☐	Dual use research of concern

Methods

n/a	Involved in the study	
☒	☐	ChIP-seq
☐	☒	Flow cytometry
☒	☐	MRI-based neuroimaging

Antibodies

Antibodies used	Primary antibodies: vinculin (Cell Signaling Technology #4650), phospho-ribosomal protein S6 Ser 235/236 (CST #4858), ribosomal protein S6 (CST #2217), phospho-p70 S6 kinase Thr389 (CST #9205), p70 S6 kinase (CST #9202), phospho-Akt (CST #4060), Akt (CST #9272), phospho-AMPK (CST #2535), AMPK (CST #2532), phospho-Chk1 Ser345 (CST #2341), phospho-Chk1 Ser345 (CST #2348), phospho-Chk2 Thr68 (CST,2197), p53 (CST #9282), and p21 (CST, #2947). Primary antibodies were used at a dilution of 1:1000 Secondary antibodies: Anti-rabbit (CST #7074), anti-mouse (CST #7076). Anti-rabbit was used at a 1:5000 dilution and anti-mouse was used at a dilution of 1:10000.
Validation	Antibodies were validated by the suppliers and by the appearance of a band at the predicted size. The manufacturers provide the following statement on their multiple approaches to antibody validation: "To ensure our antibodies will work in your experiment, we adhere to the Hallmarks of Antibody Validation™, six complementary strategies that can be used to determine the functionality, specificity, and sensitivity of an antibody in any given assay. CST adapted the work by Uhlen, et. al., ("A Proposal for Validation of Antibodies." Nature Methods (2016)) to build the Hallmarks of Antibody Validation, based on our decades of experience as an antibody manufacturer and our dedication to reproducible science." Antibodies for phospho-ribosomal protein S6 Ser 235/236 and phospho-p70 S6 kinase Thr389 were also validated by treating cells with the mTORC1 inhibitor Torin1 and observing a decrease in signal.

Eukaryotic cell lines

Policy information about cell lines

Cell line source(s)	Cell lines were obtained from ATCC (catalog numbers: A549 – CCL-185; H1299 – CRL-5803; 143B – CRL-8303; U2OS – HTB-96;

Cell line source(s)	MDA-MB-468 – HTB-132; RPE-1 – CRL-4000); A9 cells were a gift from the B. Manning laboratory (original source was ATCC, catalog number CCL-1.4)
Authentication	The identities of all cells were authenticated by satellite tandem repeat testing and referenced to ATCC values.
Mycoplasma contamination	All cell lines tested negatively for mycoplasma.
Commonly misidentified lines (See ICLAC register)	None of the cell lines used in this study are listed as commonly misidentified cell lines.

Flow Cytometry

Plots

Confirm that:

☒ The axis labels state the marker and fluorochrome used (e.g. CD4-FITC).

☒ The axis scales are clearly visible. Include numbers along axes only for bottom left plot of group (a 'group' is an analysis of identical markers).

☒ All plots are contour plots with outliers or pseudocolor plots.

☒ A numerical value for number of cells or percentage (with statistics) is provided.

Methodology

Sample preparation	Samples were prepared from cultured human cell lines. To fix cells for staining and flow cytometry, cells were trypsinized, pelleted and washed twice with PBS. Cells were resuspended in 500 µL ice-cold PBS and 5 mL ice-cold ethanol was added dropwise to each sample while vortexing in order to obtain a single-cell suspension.
Instrument	A BD FACSCanto II Cell Analyzer was used to collect data.
Software	BD FACSDiva Software was used to collect the data, and FlowJo Software was used to analyze the data.
Cell population abundance	This is not relevant because cells were not sorted.
Gating strategy	Cells were gated based on FSC and SSC to distinguish live cells for analysis.

☒ Tick this box to confirm that a figure exemplifying the gating strategy is provided in the Supplementary Information.
