# Supplementary figures and images for: Nucleotide imbalance decouples cell growth from cell proliferation
Source: Nat Cell Biol. 2022 Aug 4;24(8):1252–64. doi: 10.1038/s41556-022-00965-1 (PMC9359916; doi:10.1038/s41556-022-00965-1)

Figure 4a

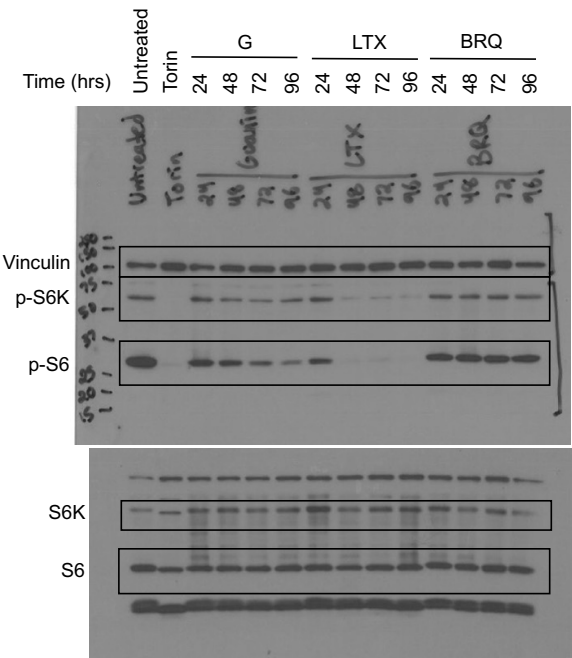

Figure 4d

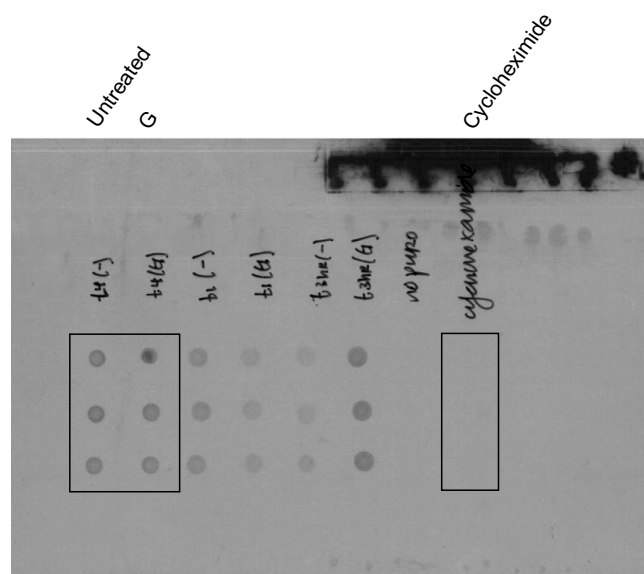

Supplement: Source Data Fig. 4 — Unprocessed western blots. [file 41556_2022_965_MOESM10_ESM.pdf]

Figure 5b

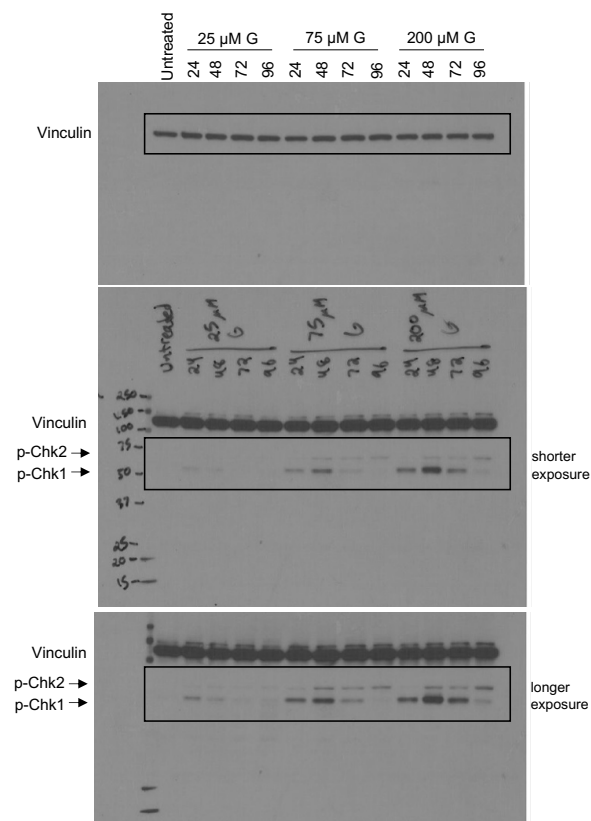

Figure 5c

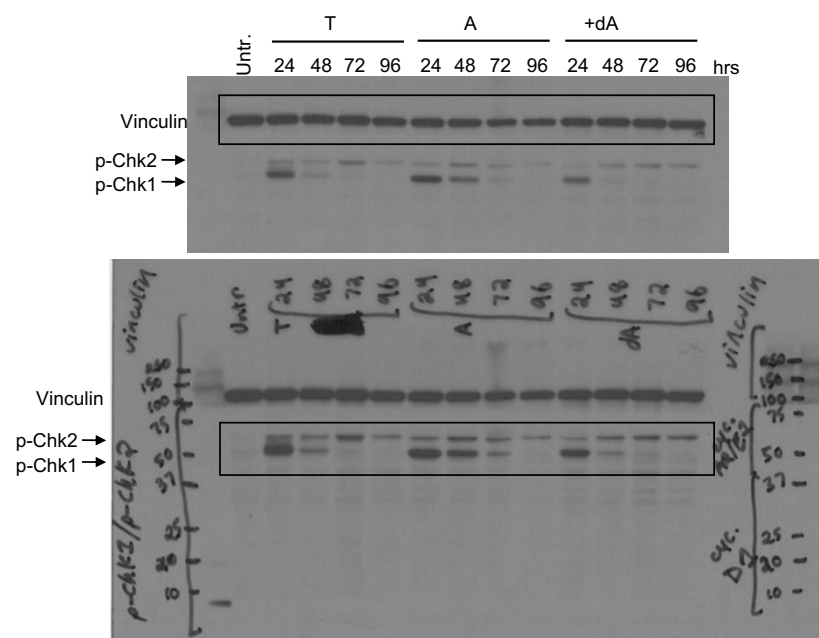

Figure 5d

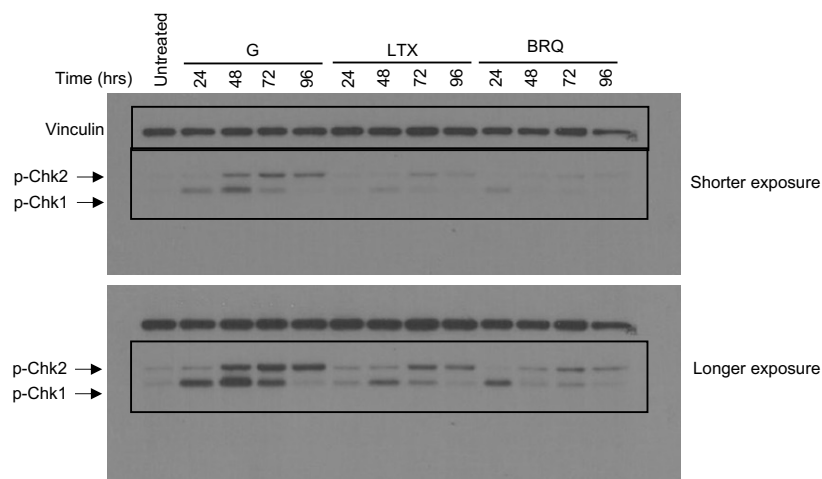

Supplement: Source Data Fig. 5 — Unprocessed western blots. [file 41556_2022_965_MOESM12_ESM.pdf]

Figure 6b

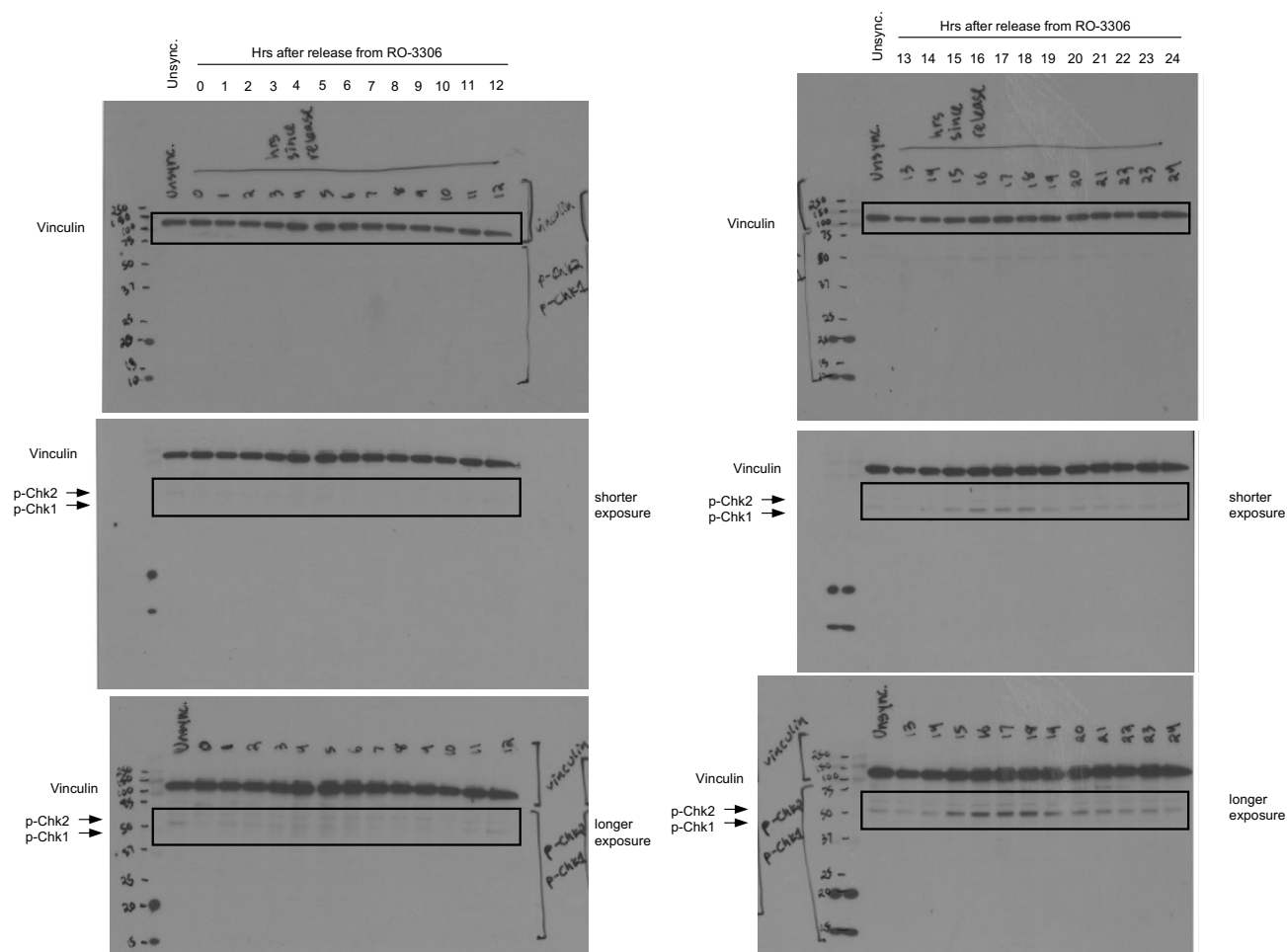

Supplement: Source Data Fig. 6 — Unprocessed western blots. [file 41556_2022_965_MOESM14_ESM.pdf]

Extended Data Figure 5a

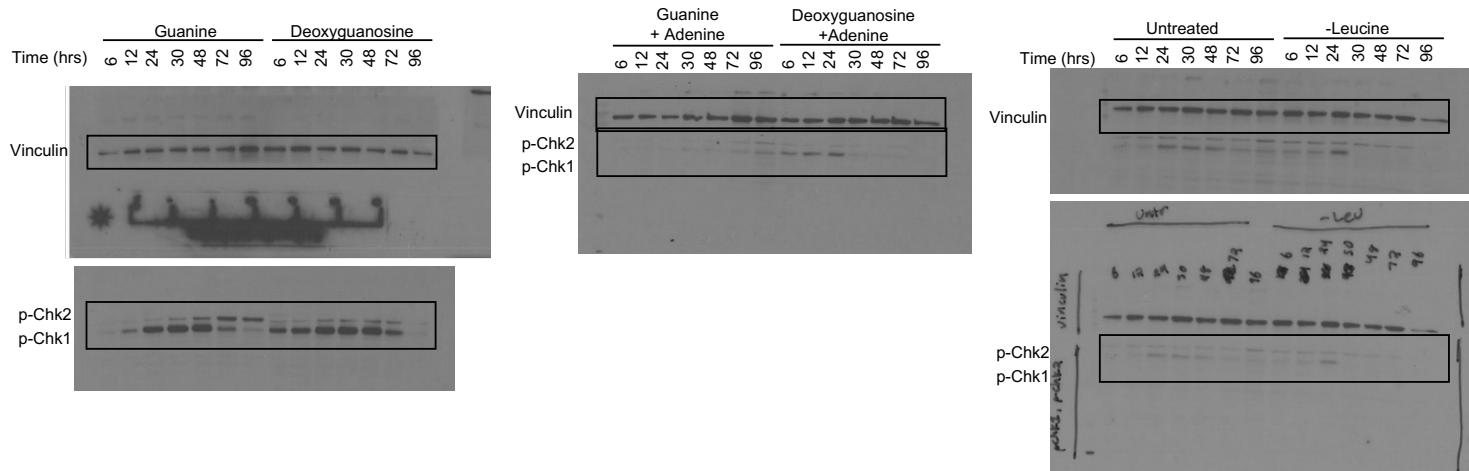

Extended Data Figure 5b

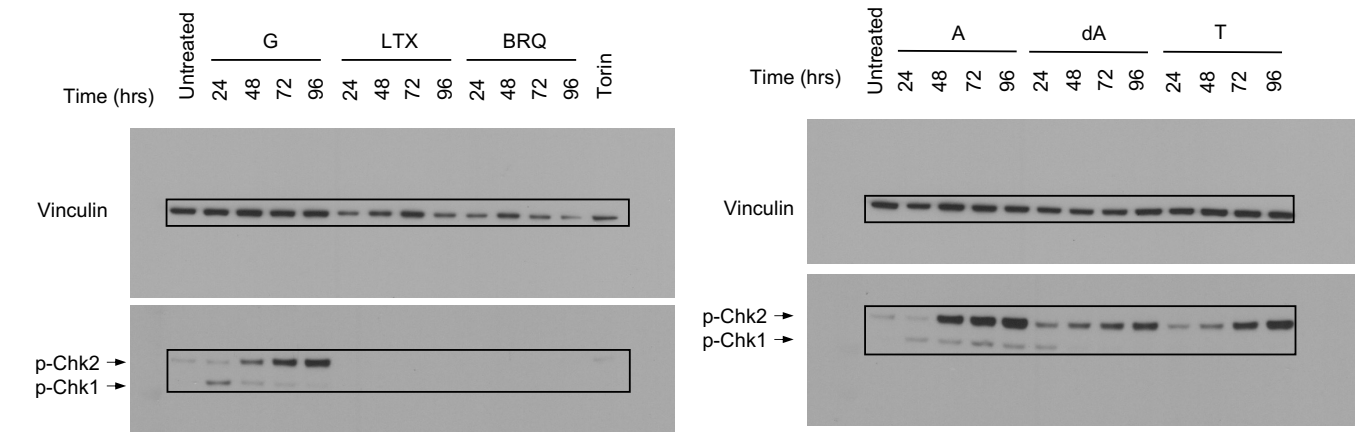

### Extended Data Figure 5c

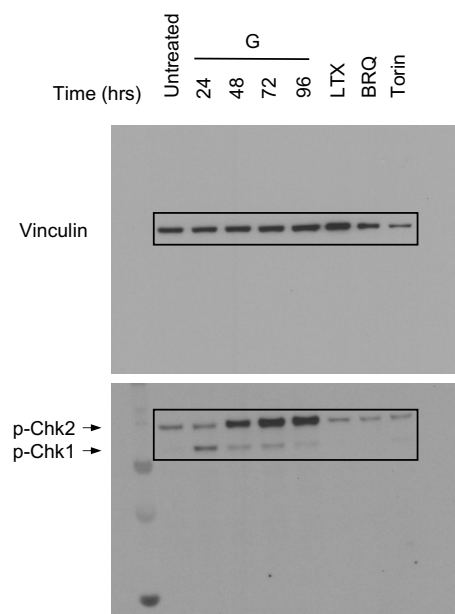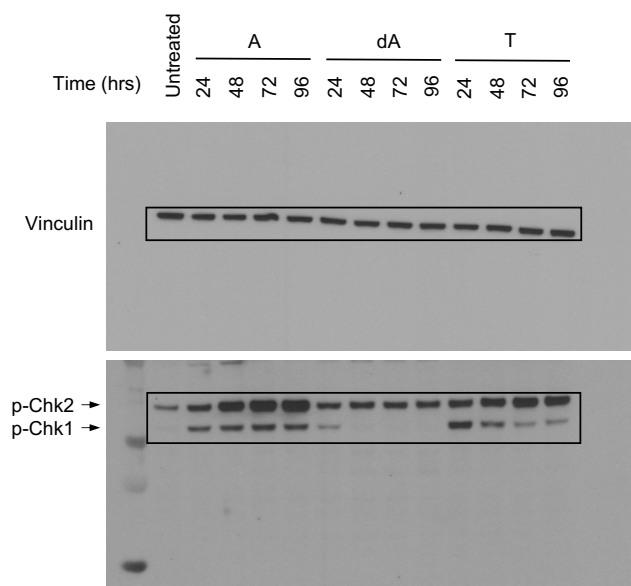

Extended Data Figure 5d

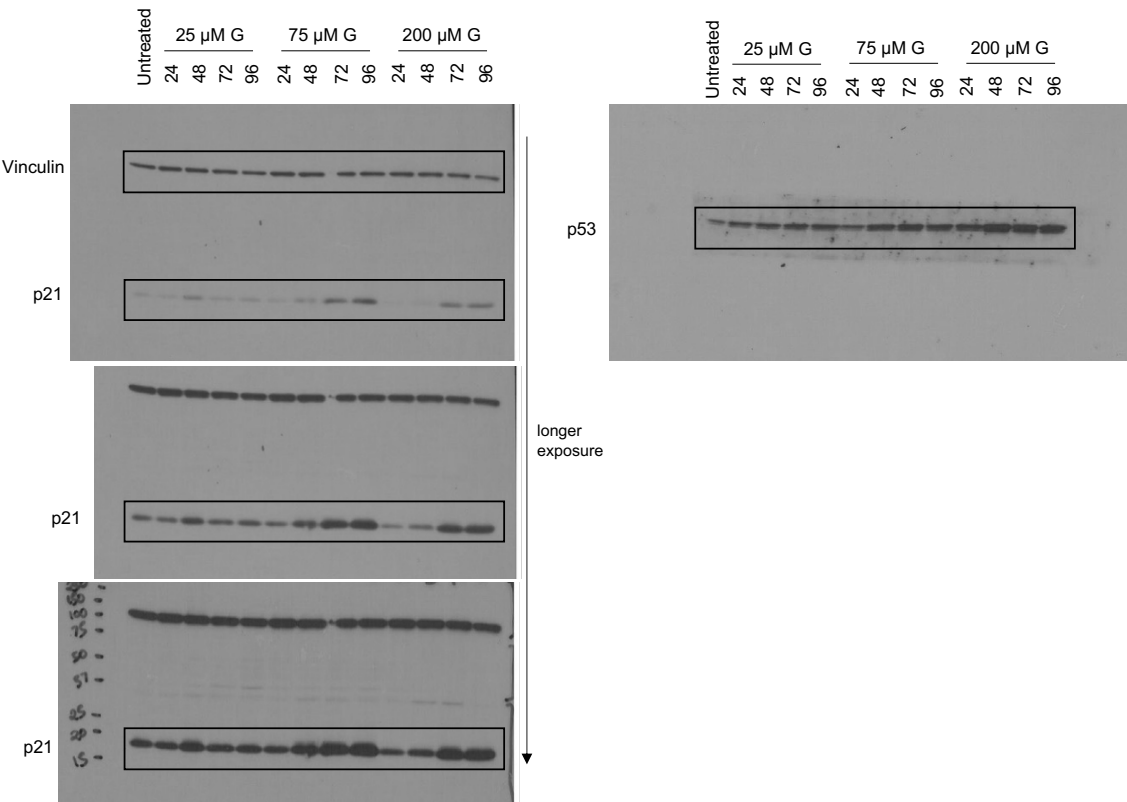

Supplement: Source Data Extended Data Fig. 5 — Unprocessed western blots. [file 41556_2022_965_MOESM21_ESM.pdf]

Extended Data Figure 6b

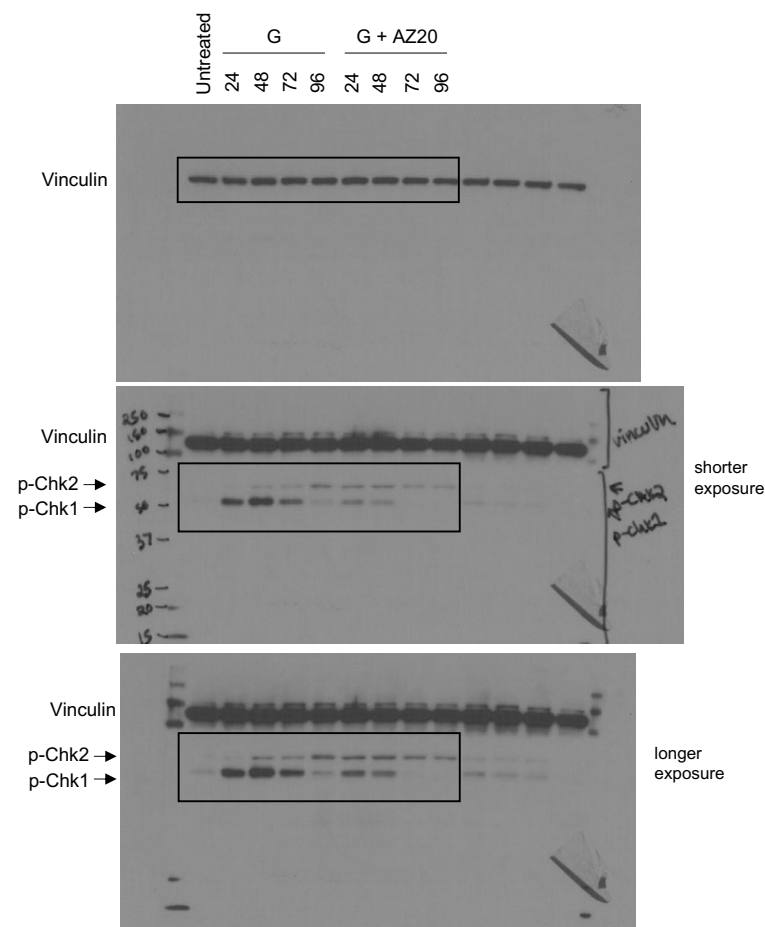

Extended Data Figure 6f

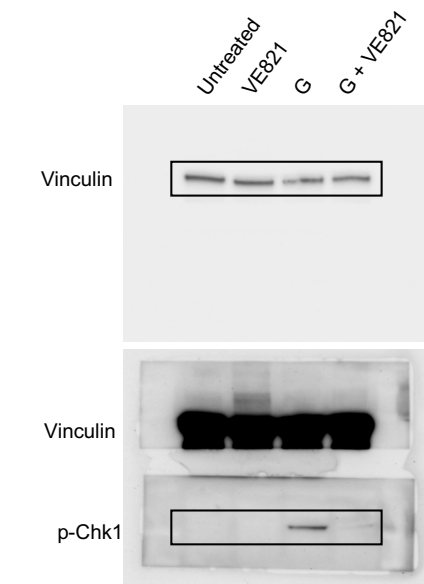

Supplement: Source Data Extended Data Fig. 6 — Unprocessed western blots. [file 41556_2022_965_MOESM23_ESM.pdf]
