# Supplementary material for: Nucleotide imbalance decouples cell growth from cell proliferation
Source: Nat Cell Biol. 2022 Aug 4;24(8):1252–64. doi: 10.1038/s41556-022-00965-1 (PMC9359916; doi:10.1038/s41556-022-00965-1)

Extended Data Figure 4a

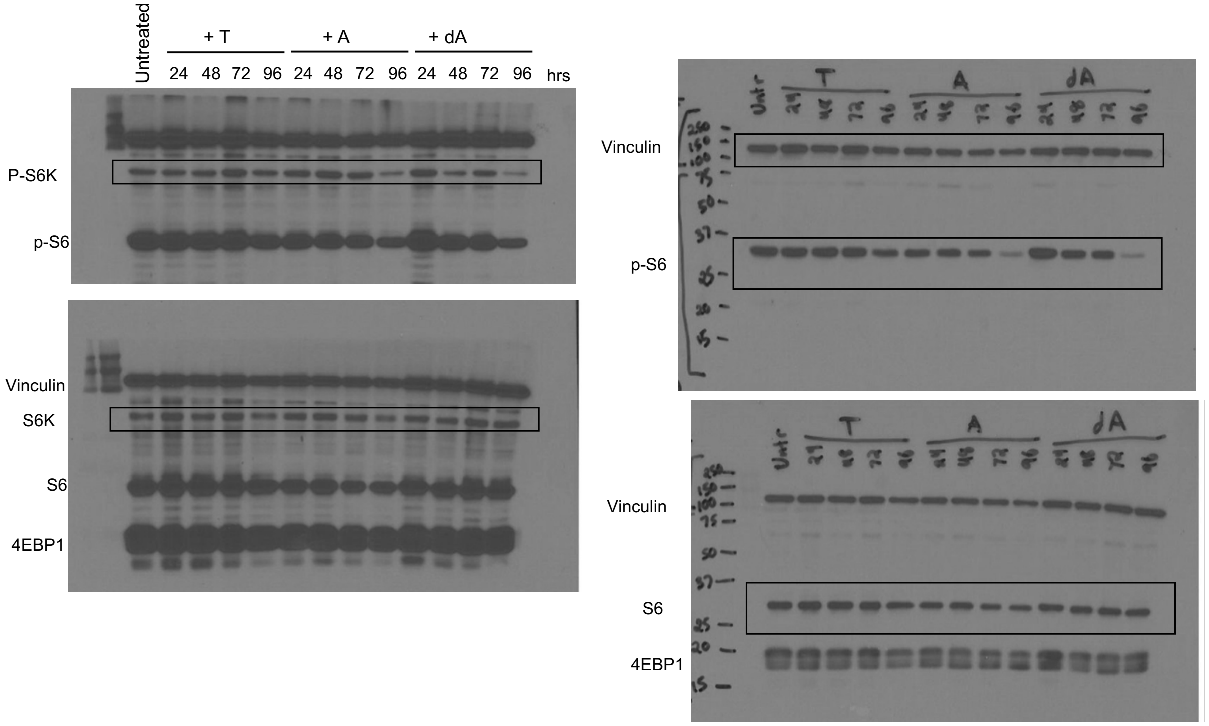

Extended Data Figure 4b

143B

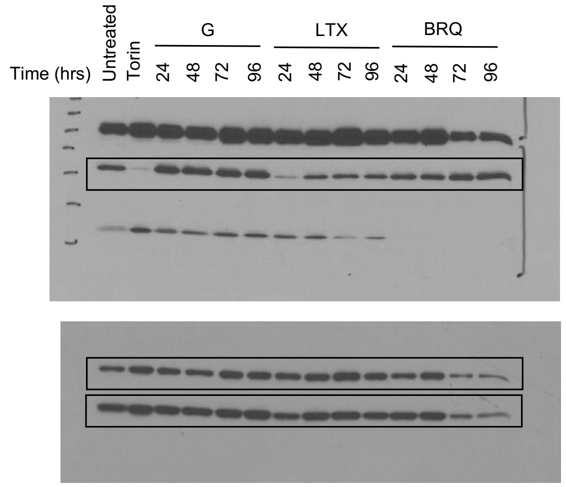

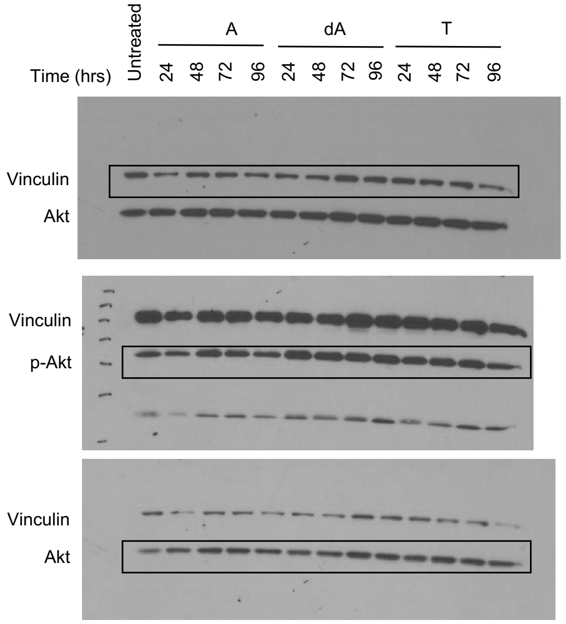

H1299

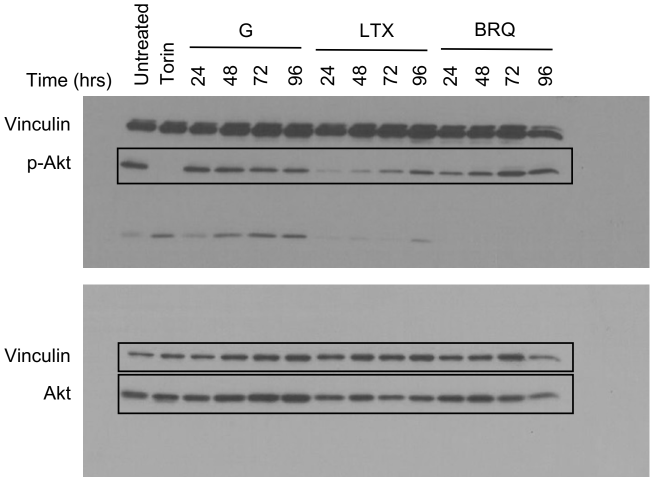

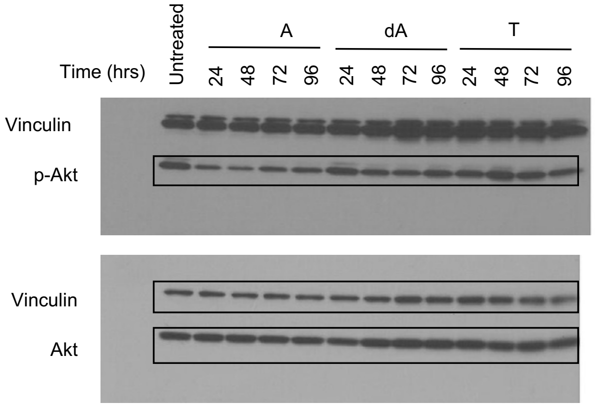

Extended Data Figure 4c

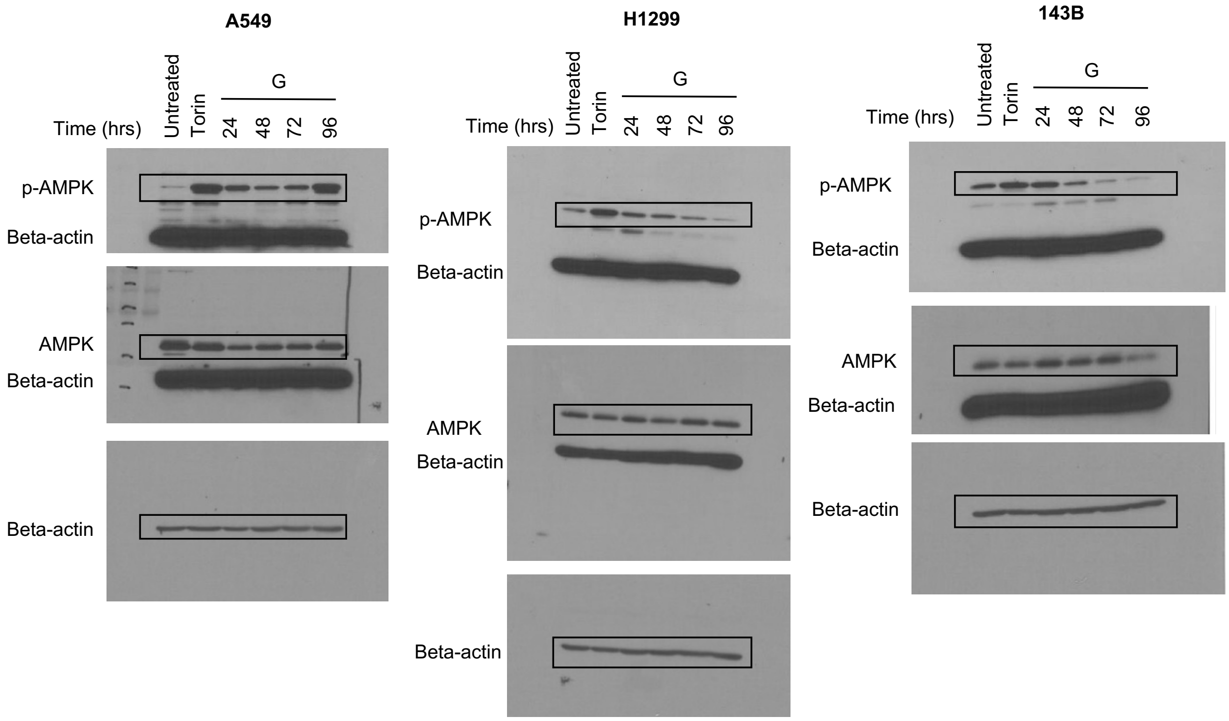

Extended Data Figure 4d

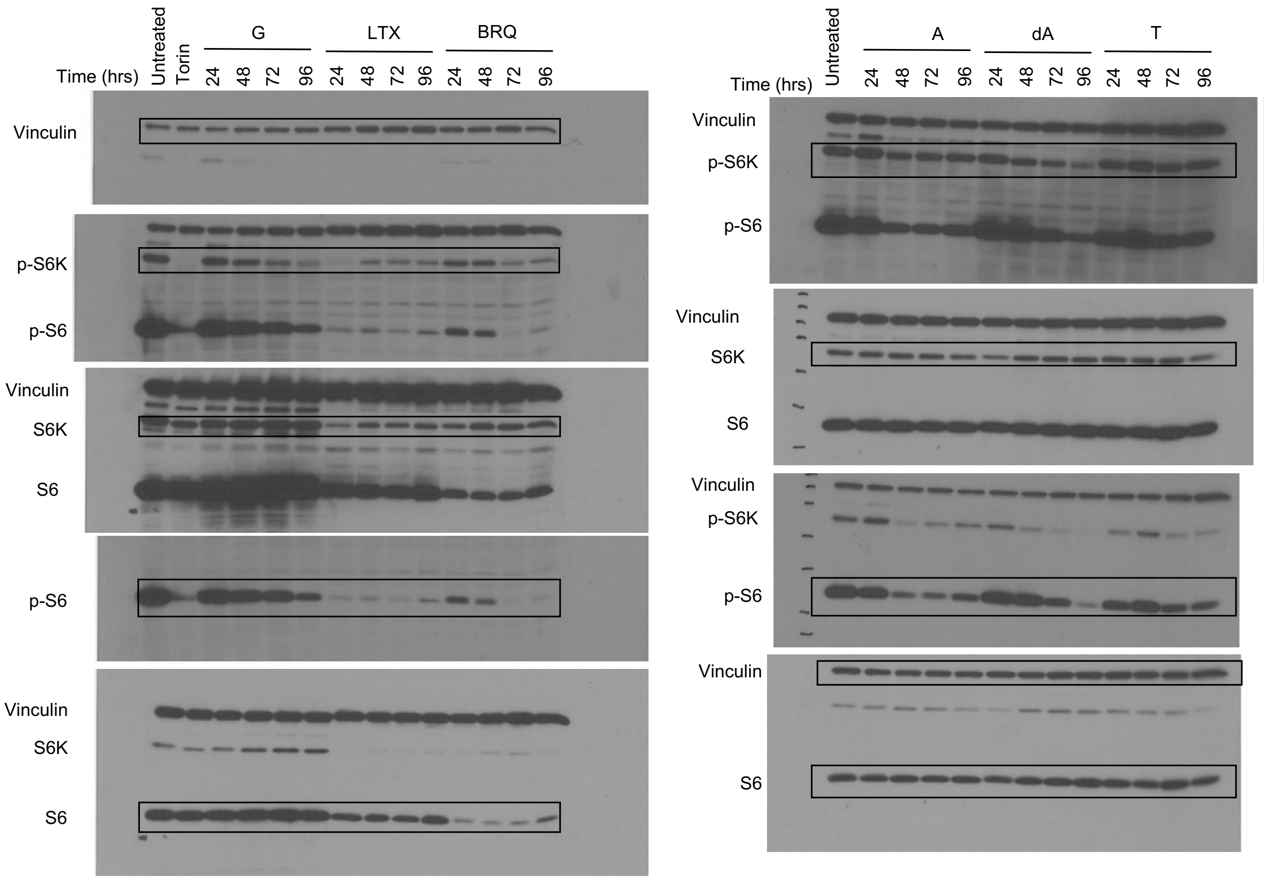

Extended Data Figure 4l

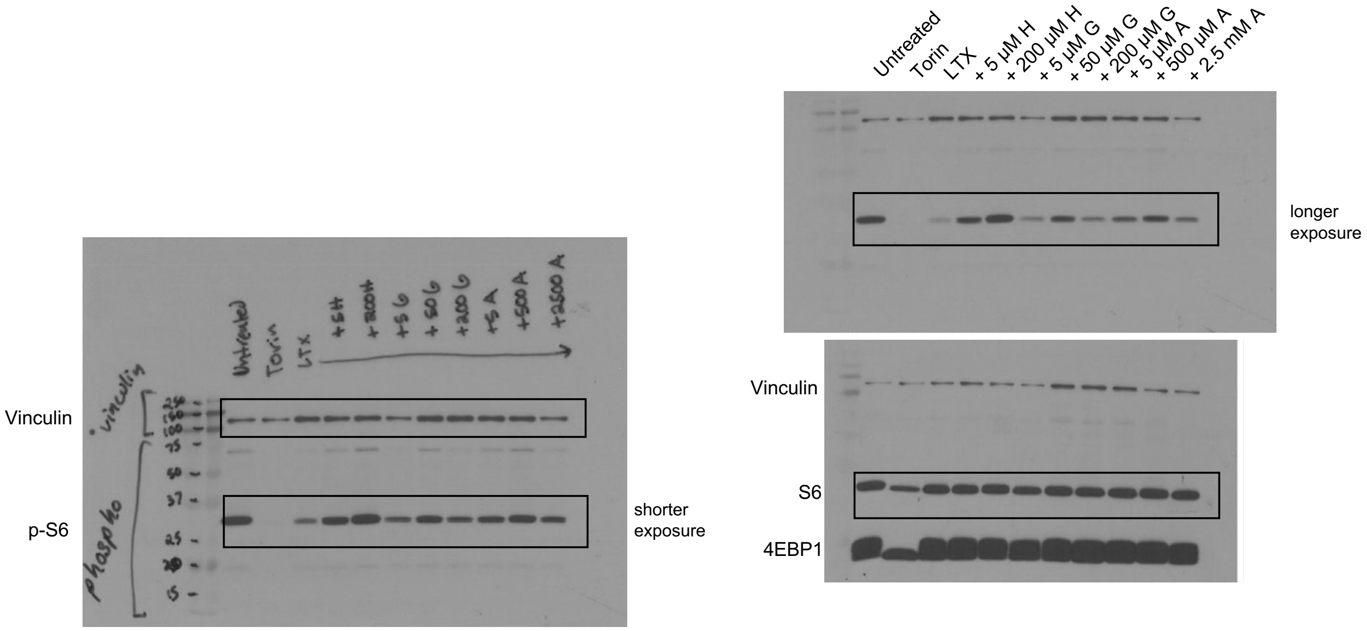

Extended Data Figure 4m

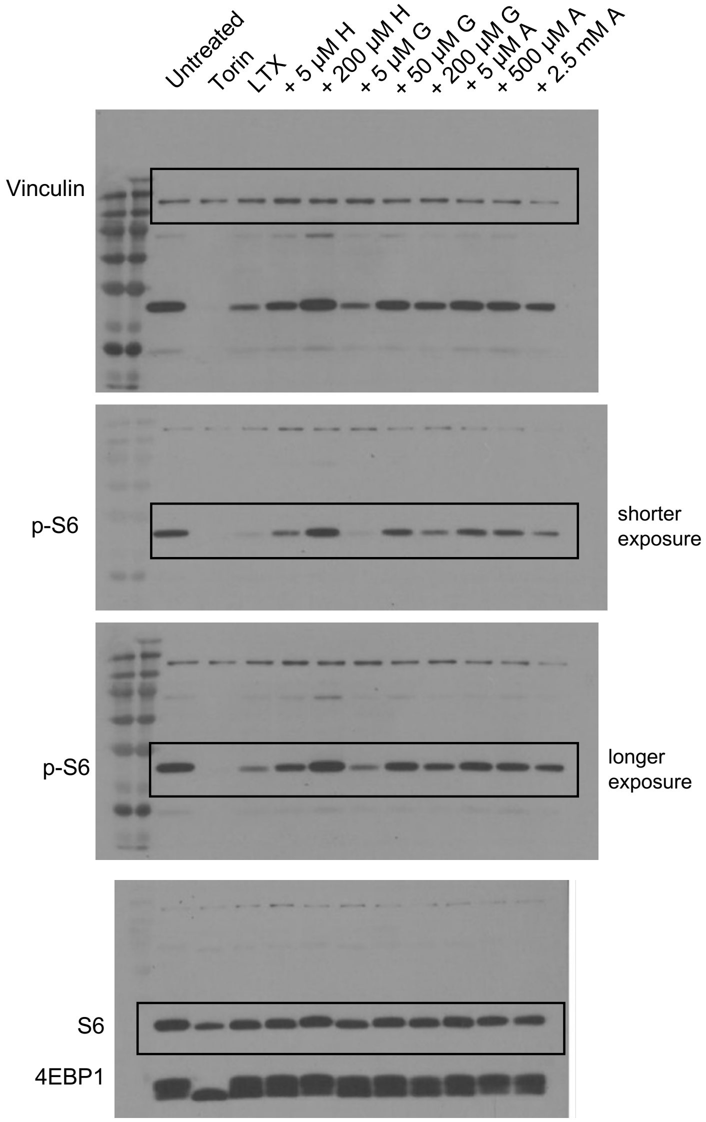

Supplement: Source Data Extended Data Fig. 4 — Unprocessed western blots. [file 41556_2022_965_MOESM19_ESM.pdf]
